# Incidence and epidemiology of acute kidney injury in a pediatric Malawian trauma cohort: a prospective observational study

**DOI:** 10.1186/s12882-020-01755-3

**Published:** 2020-03-14

**Authors:** Erica C. Bjornstad, William Muronya, Zachary H. Smith, Keisha Gibson, Amy K. Mottl, Anthony Charles, Stephen W. Marshall, Yvonne M. Golightly, Charles K. Munthali, Emily W. Gower

**Affiliations:** 1 Department of Pediatrics, Division of Nephrology, University of Alabama Birmingham, 1600 7th Avenue South, Lowder 516, Birmingham, AL 35233 USA; 2 Department of Epidemiology, University of North Carolina at Chapel Hill Gillings School of Public Health, Chapel Hill, NC USA; 3 Department of Surgery, Kamuzu Central Hospital, Lilongwe, Malawi; 4 Univeristy of North Carolina Project Malawi, Lilongwe, Malawi; 5 Division of Pediatric Critical Care Medicine, Stanford University School of Medicine, Stanford, CA USA; 6 Department of Medicine, Division of Nephrology and Hypertension, University of North Carolina, Chapel Hill, NC USA; 7 Department of Surgery, University of North Carolina, Chapel Hill, NC USA; 8 Malawi Surgical Initiative, Lilongwe, Malawi; 9 University of North Carolina Injury Prevention Research Center, Carrboro, NC USA; 10 Department of Medicine, Renal Unit, Kamuzu Central Hospital, Lilongwe, Malawi

**Keywords:** Acute kidney injury, Trauma, Africa, Pediatrics, Epidemiology

## Abstract

**Background:**

Acute kidney injury (AKI) is highly associated with mortality risk in children worldwide. Trauma can lead to AKI and is a leading cause of pediatric death in Africa. However, there is no information regarding the epidemiology of pediatric, trauma-associated AKI in Africa.

**Methods:**

Prospective cohort study of pediatric trauma patients admitted to a tertiary referral hospital in Malawi. Participants enrolled at admission were followed prospectively throughout their hospitalization. AKI was defined by creatinine-only Kidney Disease Improving Global Outcomes criteria. We calculated descriptive statistics and univariate relative risks (RR) for hypothesis-generation of potential risk factors associated with AKI.

**Results:**

We analyzed data from 114 participants. Depending on baseline creatinine definition, AKI incidence ranged from 4 to 10%. The new Schwartz equation estimated baseline creatinine values best and yielded an AKI incidence of 9.7%. Almost one in ten children died during hospitalization, but those with AKI (*n* = 4) were at significantly higher risk of death compared to those without AKI (40.0% vs 6.2%; RR 6.5, 95% CI 2.2–19.1). Burn injuries were most commonly associated with AKI (63.6%). Other potential AKI risk factors included multiple injuries, trunk or facial injuries, and recent consumption of herbal remedies.

**Conclusions:**

AKI occurs in up to 10% of admitted pediatric trauma patients in Malawi and increases the risk of death 7-fold compared to those without AKI. This large unrecognized burden in trauma requires further investment by researchers, clinicians and policymakers to develop evidenced-based triage, recognition, and management approaches to prevent the associated sequelae and potential mortality from AKI.

## Background

Estimates suggest that 13.3 million people worldwide are affected by acute kidney injury (AKI) annually, 85% of whom live in developing countries [1, 2]. Further it is estimated that up to 1.7 million deaths occur each year from AKI [1, 2]. A large meta-analysis analyzing AKI incidence globally suggests that 1 in 3 hospitalized children experience AKI [3]. Yet, no high-quality studies could be found in Africa to be included [3].

Since that meta-analysis, more studies from Africa have emerged on pediatric AKI and they have focused on infection-related AKI (e.g., AKI in those with malaria) or non-surgical-related AKI [4–6]. Trauma is also a likely risk factor. There has been a paucity of investigation into AKI associated with trauma in Africa, despite the high burden of trauma in children.

Trauma is a leading cause of morbidity and mortality throughout Africa and the leading cause of mortality worldwide for children and young adults (5–29 years of age) [7]. Organ failure, including AKI, is the third leading cause of mortality in trauma patients, after bleeding and brain injuries [8]. Traumas typical in low-resourced settings – road traffic injuries, burns, crushing in earthquakes or structural collapses – can result in AKI secondary to fluid loss, hemorrhage, and rhabdomyolysis from crush injuries. The majority of trauma-based AKI studies worldwide have looked at *critically ill* adult trauma patients and these report highly variable AKI rates, ranging 1–50% [9–14]. Though pediatric trauma studies on AKI are scarce, a California study suggests 13% of pediatric post-traumatic rhabdomyolysis patients experience AKI [15]. No studies have evaluated AKI rates amongst pediatric trauma patients in Africa. We conducted the first prospective cohort study of pediatric trauma patients in sub-Saharan Africa to investigate AKI amongst a well-established hospital-based trauma surveillance registry in Malawi. We sought to determine the incidence of AKI amongst admitted pediatric trauma patients at this single center and determine if there were any sociodemographic, injury-related, and/or clinical risk factors for those who develop AKI.

One limitation with assessing AKI in African trauma patients is the lack of baseline creatinine values and accurate urine output assessments in this population. Even large observational studies in pediatric AKI have suggested 50% do not have a known a baseline creatinine value [16]. Therefore, a secondary objective was to assess optimal methods for estimating baseline creatinine for practical implications of determining AKI in limited resource settings where only one creatinine value is obtained.

## Methods

### Design

This is a single center, prospective observational cohort study evaluating the epidemiology of AKI in admitted adult and pediatric trauma patients in Lilongwe, Malawi. This analysis included only the pediatric patients (≤18 years).

### Study setting

Kamuzu Central Hospital (KCH) and the Malawi Surgical Initiative have had a trauma registry in place for the past decade to characterize the burden of trauma in the Central Region of Malawi. KCH is the tertiary-level referral center for the Central Region, located in the capital city of Lilongwe. It has a catchment area of about 5 million people. It is the largest trauma center in the country. Acute dialysis capabilities are available, though limited. Peritoneal dialysis is available as young as 3–4 years of age; acute intermittent hemodialysis is available as young as 9–11 years of age. Modalities and access depend on age, size, and staff and resource availability at any given time.

### Pediatric population

Pediatric patients presenting to KCH for acute trauma between June and October 2019 were eligible for enrollment. Additional inclusion criteria were: age between 6 months and 18 years of age, weight > 3 kg, and expected admission > 24 h. A parent or a caregiver had to be present and provide written informed consent. Exclusion criteria included those with trauma that occurred > 5 days prior to hospital arrival and those whose primary language was not English or Chichewa (the Malawian official and national languages, respectively). All admission laboratory diagnostics had to occur within 18 h of hospital arrival. All patients were prospectively followed throughout their hospitalization.

### Outcome

The primary outcome of interest was AKI as defined by creatinine-only Kidney Disease Improving Global Outcomes (KDIGO) criteria [17]. Serum creatinine was obtained on admission and again 48–72 h later. In Malawi, it is not routine for kidney function tests or electrolytes to be collected on trauma patients, due to resource limitations. It also is not routine for urine volumes to be captured. To maximize our accuracy for diagnosing AKI, we chose to obtain two values 48 h apart to assess a change in creatinine and better apply the KDIGO AKI criteria [17]. Medical teams could obtain more laboratory tests when they deemed them medically necessary.

The University of North Carolina (UNC) Project Malawi Laboratory, a state-of-the-art laboratory on-site at KCH, performed all laboratory testing to ensure reliable and consistent laboratory results. Creatinine values were obtained on fresh serum and evaluated on Roche Cobas C311 analyzers. Creatinine was determined using Jaffe analytic methods.

A secondary outcome of interest was evaluating how best to estimate a child’s ‘baseline creatinine’ as required for interpreting the KDIGO AKI definition. Up to 50% of admitted children worldwide may not have a known baseline creatinine [16], and we would argue this number may approach 100% in some settings such as Malawi. When it is not known, a variety of approaches have been offered on how to estimate this and subsequently apply the KDIGO AKI criteria. No studies have evaluated the optimal method for estimating a baseline creatinine (or by extrapolation, estimated glomerular filtration rate (eGFR)) in an African pediatric population. A priori, we defined a normal baseline eGFR as 120 ml/min/1.73m^2^ [18].

We identified four potential definitions to estimate a child’s ‘baseline creatinine’. The first is the (1) *lowest creatinine* during admission (if 2 or more values are obtained). A priori we defined the *lowest creatinine* method as the “gold standard” on which to compare the other equation methods. This definition assigns the lowest creatinine during admission as the ‘baseline creatinine’ on which to apply KDIGO AKI criteria. If the lowest creatinine during admission is not accurate to the true baseline creatinine, then it is most likely biased away from (higher than) the true baseline due to renal dysfunction from their acute illness and subsequently would under estimate AKI in a population. In this setting, fluid resuscitation does not occur prior to hospitalization. This method is the least biased; but it requires a minimum of two creatinine values. The other three definitions rely on equations to back calculate a ‘baseline creatinine’ using patient’s height and eGFR of 120 ml/min/1.73m^2^: (2) *old Schwartz equation* (eGFR = k*height/serum creatinine, where k = 0.55 for children > 1 year of age and k = 0.45 if ≤1 year of age) [19], (3) *new (bedside) Schwartz equation* (eGFR = k*height/serum creatinine, where k = 0.413) [20], and (4) *India equation*, which is the new Schwartz equation modified for use in low-resourced settings, where k = 0.42 instead [21].

Briefly, both Jaffe and enzymatic methods are lab-based methods to determine serum creatinine. Jaffe methods are more prone to interfering substances yet are drastically cheaper than the newer enzymatic methods. The *India equation* was derived in a pediatric population of a low-resourced Indian setting where Jaffe methods were used, and *k* = 0.42 was determined to be most predictive of actual glomerular filtration rate [21]. The *old Schwartz equation* was originally based on Jaffe methods, while the *new (bedside) Schwartz equation* was originally based on enzymatic methods.

### Covariates

As this was an exploratory analysis for hypothesis generation of potential sociodemographic and clinical risk factors associated with AKI amongst pediatric trauma patients in sub-Saharan Africa, we assessed for multiple potential exposures.

Demographic variables included age in years, sex, tribal association (collapsed to Chewa versus other due to small numbers), and home district (collapsed to Lilongwe versus other due to small numbers). Because no single variable has been shown to best capture an individual’s socioeconomic status in African studies [22, 23], we obtained common variables for socioeconomic status used in other African studies: parental and child’s level of education, mother and father vital status, crowding (number of people living in the home divided by the number of rooms), type of roofing (thatch, tile, tin/iron), type of flooring (dirt, cement, tile), and possession of common items (refrigerator, television, cell phone, working vehicle, chicken(s), cow(s)).

Comorbidities were determined by patient/care-giver self-report with the exception of anemia, malaria, and sickle cell disease, which we confirmed with laboratory testing due to their potential impact on kidney function. We used the World Health Organization (WHO)‘s classification of anemia as hemoglobin< 11 g/dL [24], determined by point-of-care hemoglobin (HemoCue Hb 201+ analyzer). Sickle cell disease was determined by hemoglobin electrophoresis, and malaria by blood smears through the UNC laboratory. Human immunodeficiency virus serostatus was determined by self-report of infection or hospital-based testing and documentation as per Malawian national guidelines. Hospital disposition was defined as discharged alive, left against medical advice, or death.

Some environmental exposures are known to be nephrotoxic. Potential exposures we obtained from patient/caregiver report included: primary drinking water source, previous herbal remedies, and previous over-the-counter medications.

Trauma-related factors included type of trauma (burn, fall, road traffic injury, other), body location of trauma (head/neck, trunk, extremity, face), and multiple injuries (yes/no). We attempted to obtain a Kampala Trauma Score (a validated score for injury severity in low-resourced settings) [25] on patients, but only two patients had all variables documented in their hospital records to calculate it.

### Sample size

To estimate AKI incidence in children (≤18 years of age), we aimed to enroll 240 pediatric trauma patients admitted to KCH. This target sample size would allow us to detect a 17% incidence with precision of +/− 5%, assuming a 10% loss-to-follow-up rate for patients leaving against medical advice prior to the second creatinine value. There were no published estimates of pediatric AKI in Malawi at the time of study planning, so our estimates come from an adult study in Blantyre, Malawi of general medical admissions [26].

### Analysis

For our secondary outcome, we used descriptive analyses to assess the three different equation methods for estimating a child’s baseline creatinine compared to our a priori “gold standard” of the *lowest creatinine* method. The optimal equation method was then used to determine the incidence of AKI (our primary outcome of interest) in our study population. Choosing an equation method for baseline creatinine estimation allowed us to analyze all participants, not just those with ≥2 creatinine values. This also allows for broader clinical implications in other limited resource settings where clinicians often must make medical decisions based on only one creatinine value for assessing AKI.

We used descriptive statistics to evaluate epidemiological factors and mortality associated with those who developed AKI versus those who did not. Comparisons of sociodemographic factors, exposure history and clinical characteristics between those who did and did not develop AKI were evaluated with ANOVA and chi-square tests for categorical and continuous variables, respectively. For variables with *p*-values < 0.1, we further evaluated measures of association for AKI with univariate linear and log-linear regression models to assess potential risk factors. Sample size/outcome were too small for multivariate modelling of risk factors.

Data were double-entered into REDCap electronic data capture tools hosted at UNC at Chapel Hill [27]. All statistical analyses were conducted in SAS, version 9.4 (SAS Institute, Inc., Cary, North Carolina). The institutional review boards at UNC and Malawi’s National Health Science Research Committee approved this study.

## Results

**Fig. 1:**
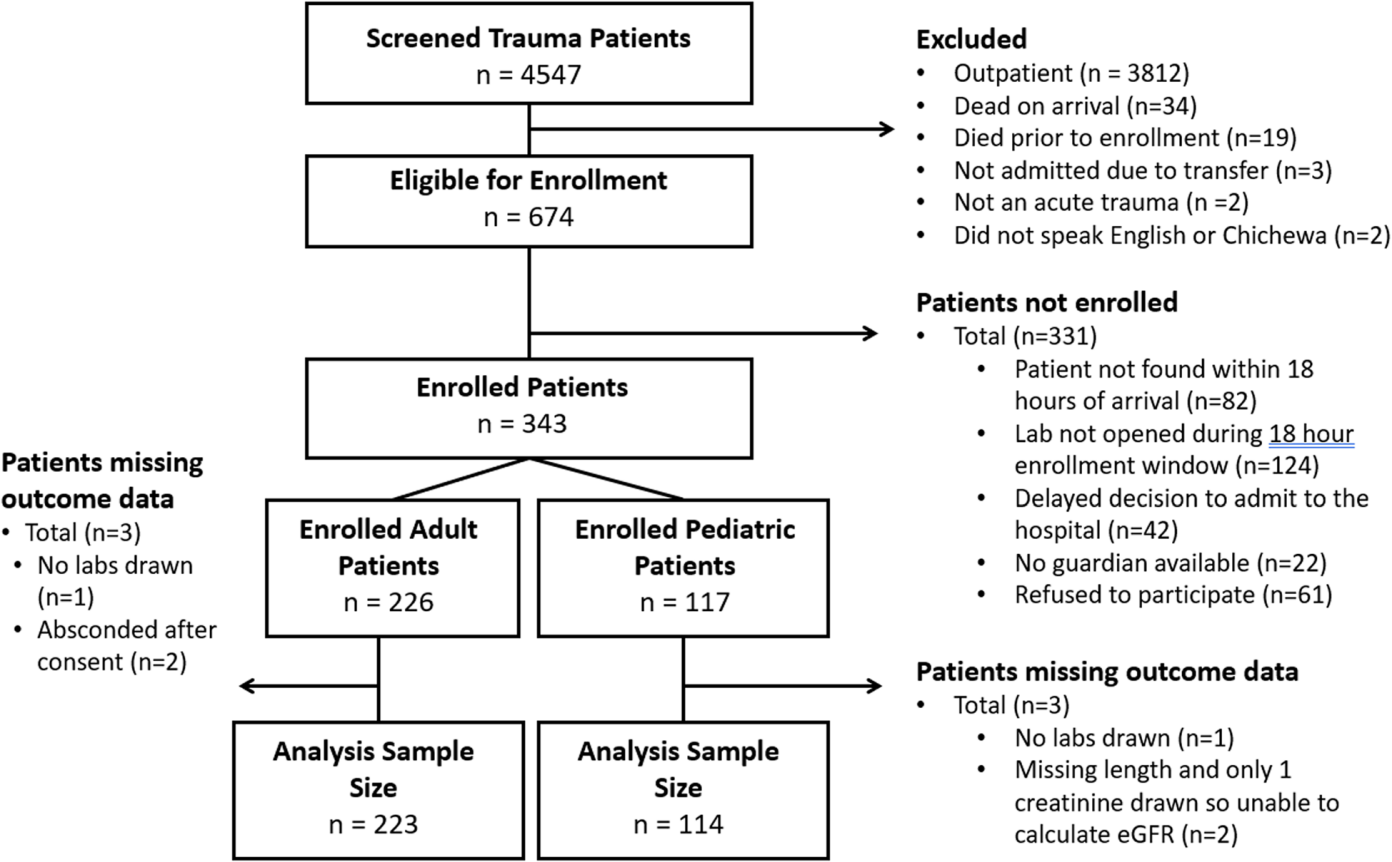
Patient enrollment flow chart. Patient enrollment flow chart for the overall study. For this analysis only the pediatric patients are analyzed

A total of 4547 adult and pediatric trauma patients presenting to KCH were screened, and 674 (14.8%) were eligible for enrollment (Fig. 1). However, only 343 (50.9%) of the eligible participants were enrolled in the overall study, primarily due to arrival times (i.e., Saturday afternoons/evenings and Sunday mornings when the laboratory was not open) and inability to find participants within the allotted enrollment time period. Analyses of those not enrolled in this study revealed no differences in types of trauma, gender, or age. A total of 114 pediatric participants were enrolled and available for analysis.

### Demographics

The majority of participants were male (62.8%) and average age was 8.1 ± 5.1 years (Table 1). Participants were primarily local, coming from within the Lilongwe district (66.4%). Nine percent of participants died (10 of 114). Overall, the participants were malnourished, a large majority had anemia (63.2%), and 16.2% had malaria. HIV status was missing for 73.7% of patients.

**Table 1:** Demographics of pediatric trauma patients admitted to Malawian Hospital by Development of AKI

	Total	AKI	No AKI	Missing
	*N* = 114	11 (9.7)	103 (90.4)	
**Age (years) (mean, STD)**	8.1 (5.1)	7.0 (6.0)	8.2 (5.0)	0
**Gender (female)**	42 (37.2)	4 (36.4)	38 (37.3)	1
**Tribe**				2
**Chewa**	74 (66.1)	8 (72.7)	66 (65.4)	
**Other**	38 (33.9)	3 (27.3)	35 (34.7)	
**District of Injury Location**				1
**Lilongwe**	75 (66.4)	8 (72.7)	67 (65.7)	
**Other District**	38 (33.6)	3 (27.3)	35 (34.7)	
**Mortality**	10 (9.0)	4 (36.4)	6 (6.0)	7^a^
**Length of Stay**^**b**^**(days) (median, IQR)**	12 (7, 26)	19.5 (9, 35)	12 (7, 25)	7^a^
**Time of Presentation to Hospital**				
**Injury occurred < 24 h prior to admission**	84 (74.3)	9 (81.8)	75 (73.5)	1
**Day of the week (weekend, Saturday-Sunday)**	12 (10.5)	2 (18.2)	10 (9.7)	0
**Time of Day (daytime, 08:00–16:00)**	42 (37.5)	6 (54.6)	36 (35.6)	2
**Comorbidities**				
**Anemia**	72 (63.2)	7 (63.6)	65 (63.1)	0
**Malaria**	18 (16.2)	1 (9.1)	17 (17.0)	3
**Sickle Cell Disease**				4
**Sickle Cell Disease**	0 (0)	0 (0)	0 (0)	
**Sickle Cell Trait**	5 (4.6)	0 (0)	5 (5.1)	
**Normal Hemoglobin**	105 (95.5)	11 (100)	94 (95.0)	
**Malnutrition**^**c**^**(median, IQR)**				
**Weight-for-age Z-score**^**d**^	−0.3 (−1.4, 0.3)	−1.7 (−1.7, −1.7)	−0.2 (−1.3, 0.5)	85
**Height-for-age Z-score**	− 0.9 (− 1.9, 0.3)	− 0.8 (−2.2, 0.2)	−0.9 (− 1.9, 0.3)	7
**Weight-for-length Z-score**	−0.3 (− 1.2, 0.4)	− 0.6 (− 2.1,1.0)	−0.3 (− 1.2, 0.4)	69
**Past Medical History** **(self-report)**				0
**Prematurity**	2 (1.9)	0 (0)	2 (2.1)	7
**Seizures**	5 (4.4)	1 (9.1)	4 (3.9)	0
**Asthma**	1 (0.9)	0 (0)	1 (1.0)	0
**None**	108 (94.7)	10 (90.9)	98 (95.1)	0

All expressed as N and column percent except where specified

KDIGO criteria used to define AKI and new Schwartz equation estimated baseline creatinine

*AKI* Acute kidney injury, *IQR* interquartile range, *STD* standard deviation

^a^4 patients absconded, 3 patients missing files

^b^Determined only for patients discharged alive

^c^Z-scores obtained using WHO AnthroPlus software, 2007 WHO reference data

^d^Weight-for-age Z scores are only provided for children up to age 10 years, WHO does not provide referent values after 10 years of age

### Outcome

**Fig. 2:**
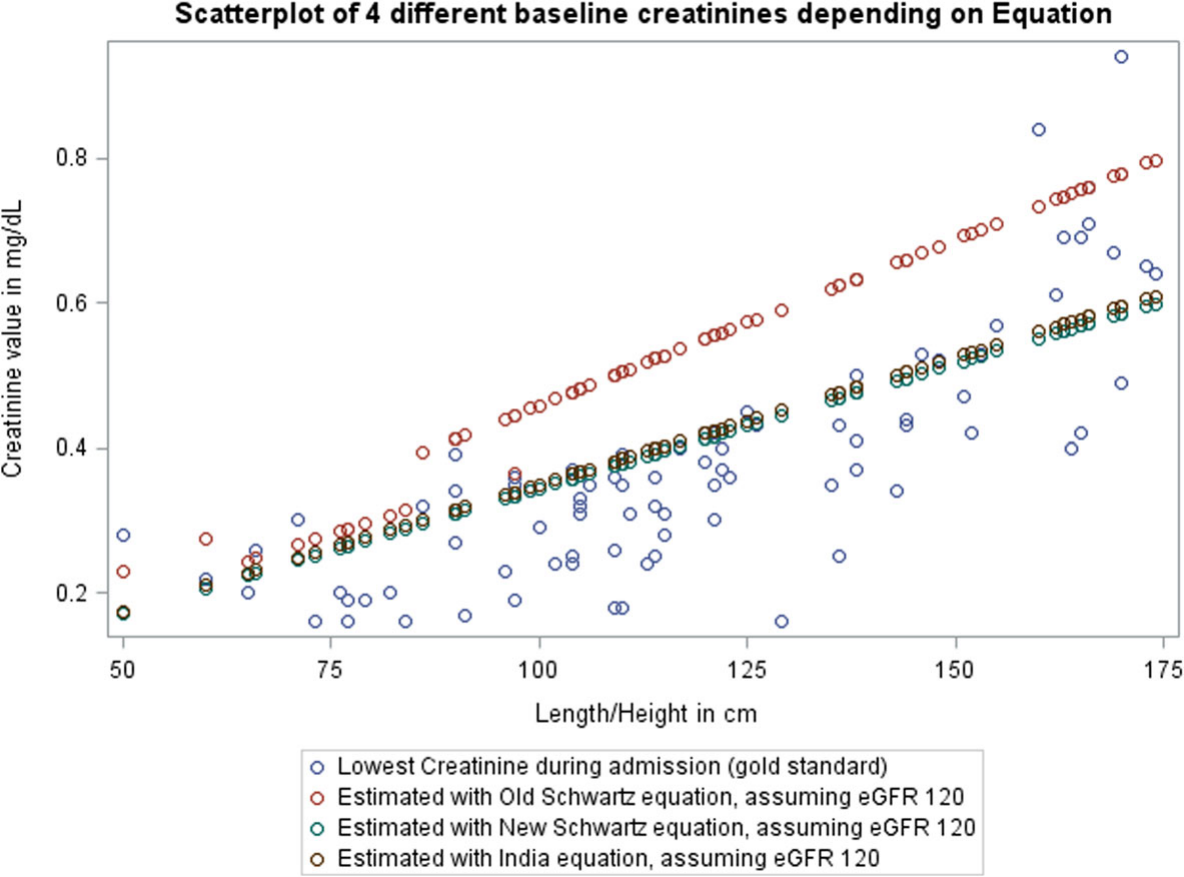
Scatterplot of baseline creatinine estimation by child’s length/height using four different methods. Only patients who had 2 creatinine values were used for this analysis, since the method using the lowest creatinine during admission requires a minimum of two values. eGFR = estimated glomerular filtration rate

The incidence of AKI varied depending on the method used to estimate a child’s “baseline creatinine.” To determine the equation method that estimated baseline creatinine closest to the *lowest creatinine method* (a priori determined “gold standard”), we initially limited the analyses to those who had two creatinine values. The estimated incidences were the same for all methods (8.8%) except the *old Schwartz* eq. (4.4%) (Table 2). Figure 2 provides a visualization for the estimated baseline creatinine using the four different methods. The best approximator to the lowest creatinine value appears to be the *new (bedside) Schwartz* equation using the original kappa (0.413) or the kappa (0.42) (India equation). For ease of use and consistency with other literature, we used the *new Schwartz* equation for further analyses. We conducted a sensitivity analysis with kappa = 0.42 and found no differences in our results.

**Table 2:** Incidence of AKI by estimated baseline creatinine method^a^

AKI Definition	AKI #	AKI %	AKI Stage 2 or 3%
Absolute 0.3 change or ≥ 1.5 rise of one of:	–	–	–
(1) lowest creatinine (“gold standard”)	8 of 91	8.8%	2.2% (*n* = 2)
(2) baseline creatinine estimated by Old Schwartz [19]	4 of 91	4.4%	1.1% (*n* = 1)
(3) baseline creatinine estimated by New Schwartz [20]	8 of 91	8.8%	1.1% (*n* = 1)
(4) baseline creatinine estimated by India equation (modified New Schwartz for low-resourced settings) [21]	8 of 91	8.8%	1.1% (*n* = 1)

^a^Restricted to those with 2+ creatinine values, *n* = 91 of 114

When using all available data (i.e., including participants with one or more creatinine value(s)), the *new Schwartz* equation estimates an AKI incidence of 9.7%. The *old Schwartz* equation estimated the lowest incidence (5.3%). The average incidence using all four approaches was 8.4%.

### Mortality

A total of 10 children died prior to hospital discharge. The risk of death was much higher (relative risk (RR) 6.5, 95% confidence interval (CI) 2.2–19.1) for those who developed AKI (4/10, 40.0%) than those who did not develop AKI (6/97, 6.2%).

### Length of stay

Participants who survived to discharge were hospitalized a median of 12 days after admission (interquartile range (IQR) 7–26) (Table 1). Participants with AKI tended to have a longer length of stay (19.5 days, IQR 9–35) compared to those without AKI (12 days, IQR 7–25).

### Potential risk factors

A third of participants had burns (33.3%) and falls (30.6%), yet two-thirds of participants who developed AKI had burn injuries (63.6%) (Table 3). Trauma-related factors that were associated with AKI included burn injuries, multiple injuries (versus a single injury), trunk and facial injuries (Table 4).

**Table 3:** Trauma-related and nephrotoxic exposure-related factors amongst admitted pediatric trauma patients in Malawi by Development of AKI

	Total	AKI	No AKI	Missing
	*N* = 114	11 (9.7)	103 (90.4)	
**Type of Trauma**				6
**Burn**	36 (33.3)	7 (63.6)	29 (29.9)	
**Fall**	33 (30.6)	2 (18.2)	31 (32.0)	
**Road Traffic Injury**	24 (22.2)	2 (18.2)	22 (22.7)	
**Other**	15 (13.9)	0 (0)	15 (15.5)	
**Primary Location of Trauma**				1
**Head/Neck**	21 (18.6)	1 (9.1)	20 (19.6)	
**Trunk**	23 (20.4)	4 (36.4)	19 (18.6)	
**Extremity**	56 (49.6)	3 (27.3)	53 (52.0)	
**Face**	13 (11.5)	3 (27.3)	10 (9.8)	
**Multiple Injuries**	56 (49.1)	8 (72.7)	48 (46.6)	0
**All Trauma Locations**^**a**^				1
**Head/Neck**	31 (27.4)	3 (27.3)	28 (27.5)	
**Trunk**	38 (33.6)	6 (54.6)	32 (31.4)	
**Extremity**	81 (71.7)	8 (72.7)	73 (71.6)	
**Face**	23 (20.4)	4 (36.4)	19 (18.6)	
**Drinking Water Source**				3
**River/Lake**	6 (5.4)	0 (0)	6 (6.0)	
**Community Pipe/Bore hole**	79 (69.3)	7 (63.6)	72 (69.9)	
**Piped (Exterior)**	17 (15.3)	3 (27.3)	14 (14.0)	
**Piped (Interior)**	9 (8.1)	1 (9.1)	8 (8.0)	
**Medications taken in previous 7 days**	34 (29.8)	3 (27.3)	31 (30.1)	0
**Herbal remedies taken in previous 7 days**	4 (3.5)	2 (18.2)	2 (1.9)	0

All expressed as N and column percent except where specified

Categories are mutually exclusive except where specified

KDIGO criteria used to define AKI and new Schwartz equation estimated baseline creatinine

*AKI* Acute kidney injury

^a^Multiple categories allowed

**Table 4:** Potential AKI risk factors in admitted pediatric trauma patients in Malawi

Exposure	Total	AKI Episodes	Crude Risks	Risk Differences	95% CI	Relative Risks	95% CI
**Burn Injury**	36	7	19.4% (8.2–36.0%)	13.9%	−0.1 to 27.9	3.5	1.1 to 11.2
**Non-burn Injury**	72	4	5.6% (1.5–13.6%)	Reference		Reference	
**Multiple Injuries**	56	8	14.3% (6.4–26.2%)	9.1%	−1.7 to 19.9	2.8	0.8 to 9.9
**Single Injury**	58	3	5.2% (1.1–14.4%)	Reference		Reference	
**Primary Location of Trauma**^**a**^							
**Head/Neck**	21	1	4.8% (0.1–23.8%)	−0.6%	− 11.5 to 10.3	0.9	0.1 to 8.1
**Trunk**	23	4	17.4% (5.0–38.8%)	12.0%	−4.5 to 28.6	3.2	0.8 to 13.4
**Face**	13	3	23.1% (5.0–53.8%)	17.7%	−5.9 to 41.4	4.3	1.0 to 19.0
**Extremity**	56	3	5.4% (1.1–14.9%)	Reference		Reference	
**Any Trunk Injury**	38	6	15.8% (4.2–27.4%)	9.1%	−3.8 to 22.0	2.4	0.8 to 7.3
**Non-trunk Injuries**	75	5	6.7% (1.0–12.3%)	Reference		Reference	
**Herbal remedies taken in previous 7 days**	4	2	50.0% (6.8–93.2%)	41.8%	−7.5 to 91.1	6.1	1.9 to 19.6
**None**	110	9	8.2% (4.9–16.6%)	Reference		Reference	

KDIGO criteria used to define AKI and new Schwartz equation estimated baseline creatinine

*AKI* Acute kidney injury, *CI* confidence interval

^a^Category is mutually exclusive

We evaluated the potential for concurrent exposures to increase one’s risk of developing AKI, including drinking water source, medications and herbal remedies prior to arrival, and iodinated contrast (Table 3). Only two children had computed tomography exams (neither with iodinated contrast). Amongst concurrent potentially nephrotoxic exposures, only herbal remedies taken within the preceding seven days was identified as a potential risk factor (RR 6.1, 95% CI 1.9–19.6) (Table 4), but this result should be viewed with caution as only four patients received herbal remedies prior to arrival.

We found no trends for a variety of socioeconomic status indicators impacting AKI risk (Table 5).

**Table 5:** Socioeconomic status factors amongst admitted pediatric trauma patients in Malawi by presence of AKI

	Total	AKI	No AKI	Missing
	*N* = 114	11 (9.7)	103 (90.4)	
**Education level completed in years (median, IQR)**				
**Patient**	2 (0, 4)	1 (0, 5)	2 (0, 4)	10
**Mother**	6 (4, 10)	5 (2, 10)	6 (4, 10)	13
**Father**	8 (4, 10)	6.5 (0, 10)	8 (4, 10)	21
**Crowding factor**^**a**^**(median, IQR)**	1.5 (1.2, 2.0)	1.5 (1.2, 2.0)	1.5 (1.2, 2.0)	2
**Type of Roof**				0
**Thatch**	45 (39.5)	5 (45.5)	40 (38.8)	
**Tin/Iron**	69 (60.5)	6 (54.6)	63 (61.2)	
**Type of Floor**				1
**Dirt**	60 (53.1)	6 (54.6)	54 (52.9)	
**Cement**	53 (46.9)	5 (45.5)	48 (47.1)	
**Parent(s) deceased**^**b**^				
**Mother**	4 (3.6)	0 (0)	4 (4.0)	
**Father**	10 (9.1)	0 (0)	10 (10.1)	
**Both**	4 (3.6)	0 (0)	4 (4.0)	
**Possessions**^**b**^				
**Refrigerator**	13 (11.5)	2 (18.2)	11 (10.8)	1
**Television**	26 (23.2)	3 (27.3)	23 (22.8)	2
**Cell Phone**	88 (78.6)	8 (72.7)	80 (79.2)	2
**Agricultural Land**	58 (52.3)	5 (50.0)	53 (52.5)	3
**Working vehicle**	6 (5.4)	0 (0)	6 (6.0)	3
**Cow(s)**	5 (4.5)	0 (0)	5 (5.0)	2
**Chicken(s)**	46 (41.1)	2 (18.2)	44 (43.6)	2
**Goat(s)**	21 (18.8)	2 (18.2)	19 (18.8)	2
**Bicycle**	40 (36.0)	3 (27.3)	37 (37.0)	3
**Ox Cart**	4 (3.6)	0 (0)	4 (4.0)	2

All expressed as N and column percent except where specified

Categories are mutually exclusive except where specified

KDIGO criteria used to define AKI and new Schwartz equation estimated baseline creatinine

*AKI* Acute kidney injury, *IQR* interquartile range

^a^Crowding factor is number of people living in a home divided by number of rooms in the home

^b^Categories are not mutually exclusive

## Discussion

To our knowledge, this is the first AKI study in African pediatric trauma patients. The incidence of AKI was 9.7% amongst admitted pediatric trauma patients at this single center in Malawi. Only a few AKI studies in trauma patients have occurred in Africa, but all are in adult patients and were performed in South Africa [28–31]. Our estimate that 10% of pediatric trauma patients have AKI is less than the incidence found in one of the adult studies in Durban, South Africa, of critically-ill trauma patients (15%) [29], but it is higher than the AKI incidence of adult trauma patients (critically-ill and non-critically ill) on presentation in Pietermaritzburg, South Africa (5.6%) [30]. Our findings seem to be a reasonable estimate of AKI in pediatric trauma patients in Malawi, yet further studies should evaluate if this incidence is similar in other regions throughout Africa.

Our study is unique as it is the first in pediatric trauma in Africa, a high-risk group of patients. A previous meta-analysis on pediatric AKI incidence worldwide found a much higher incidence (33%) than our study [3]. However, these children were from high-income settings and likely of higher acuity. Only two studies included in the meta-analysis occurred in low- or low-middle income countries and none occurred in Africa. Higher-income settings can screen for AKI daily, which is not a reality throughout Malawi or many other sub-Saharan African countries. Routine screening to monitor for AKI remains uncommon in low-resourced areas. It is also possible that, once hospitalized, children in higher-income settings are exposed to additional medical nephrotoxins that are not available in Malawi (i.e., iodinated contrast, certain nephrotoxic antimicrobials). The AKI incidence in our cohort mirrors that of a recently published cohort of hospitalized children in Blantyre, Malawi (11%) [32]. These discrepancies of AKI incidence highlight the need for additional robust epidemiological studies of AKI in Malawi and other sub-Saharan African countries as well as the inclusion of such sites in future efforts to understand and improve screening and management efforts for pediatric AKI worldwide.

AKI is a well-known risk factor for mortality in children with sepsis, malaria, and critical illness [4–6, 16, 33, 34]. AKI in adult trauma patients is also known to increase the risk of death [35–39], but little is known about AKI in pediatric trauma patients. To our knowledge, this is only the third study to show that AKI in children with trauma significantly increased the risk of death compared to those without AKI (40% vs 6.5%, RR 6.5 with 95% CI 2.2–19.1). A previous retrospective study in 88 pediatric critically-ill trauma patients in the United States (U.S.) who were also intubated saw an increase in mortality with AKI (23% mortality in those with AKI versus 5.5% mortality in those without AKI) [40]. Another U.S. retrospective study in children with burns only (*n* = 119) saw higher mortality in those with AKI than those without AKI (8.9% vs 1.5%) [41].

Given the limited knowledge about the AKI risk in pediatric trauma patients, little is also known about potential risk factors. What is known about trauma-related AKI risk factors is drawn from the adult literature. The potential risk factors we identified in our study (multiple injuries and burns) have also been shown in adult patients. Several studies have identified high rates of AKI in adult burn patients [35–37, 42, 43]. The retrospective study on pediatric burn patients also found a high incidence of AKI and that development of sepsis was an independent risk factor for AKI [41]. We did not evaluate for sepsis in our study due to the additional associated costs.

In addition, we found that trunk and facial injuries as well as consumption of herbal remedies were potential risk factors for AKI in trauma patients. These have not been seen in other studies of trauma patients, but it is also possible these were not evaluated in prior studies. A large multicenter study in France found that hemorrhagic shock and associated hypoperfusion laboratory values were associated with a higher risk of AKI [8], yet these are not evaluations readily available in Malawi, nor in many low-resourced areas. Trunk injuries seem like a reasonable risk factor given the anatomical location of the kidneys in the trunk. Similarly, herbal remedies are known nephrotoxins, so it also is plausible for this to be a risk factor. However, all of these potential risk factors (and potentially others we did not explore such as sepsis) deserve further investigations with larger sample sizes as the study was powered for determining the incidence of AKI.

When we evaluated only patients surviving to discharge, we found a trend towards a longer length of stay in patients with AKI versus those without (19.5 days vs 12 days). While this trend lacked statistical significance, the large difference in our small patient population suggests that further investigation in a larger study powered to detect these differences is warranted. Longer hospitalizations are seen in adult AKI trauma studies [9, 44]. If this trend is also reflected in pediatric trauma patients, this is potentially an outcome measure that needs continued study as this impacts not only the patients’ health, but also the financial impact on healthcare systems and families.

### AKI definitions

An essential component to AKI assessment according to KDIGO criteria is knowing a child’s healthy baseline creatinine value. No pediatric study in Africa has evaluated the optimal method for estimating a child’s baseline creatinine when it is not known. Zappitelli et al., performed a retrospective analysis in the U.S. and compared baseline creatinine estimations with true known values to assess which method performed best [18]. Ideally, we would have been able to do something similar, but no patients in our study had a known baseline creatinine value, which is common in Malawi and other low-resourced areas. Zappitelli et al., and others have shown that depending on how one defines AKI, the incidences will vary [18, 45]. Up to 50% of admitted children will not have a known baseline creatinine and some method will be employed on how to define this baseline in order to apply the KDIGO AKI criteria [16, 18, 46, 47]. To our knowledge, this is one of the first studies in Africa to explore different definitions for AKI in children within the KDIGO framework.

Building upon previous work, we assumed a baseline eGFR of 120 ml/min/1.73m^2^ and evaluated several different equations to subsequently back-calculate a baseline creatinine. We compared these equations to our a priori defined gold standard of ‘lowest creatinine’ during hospitalization. Using the *new Schwartz equation* seemed the most appropriate in this population for several reasons. First, the new Schwartz equation allowed us to include all patients, even if only one creatinine value was obtained. Obtaining one creatinine value is more realistic for other low-resourced settings, where it may be a struggle to obtain multiple creatinine values (required for using the *lowest creatinine* method). Second, visual inspection of all estimated creatinine values based on height (Fig. 2) demonstrated that new Schwartz outperformed old Schwartz, particularly after one year of age when the constant changes in the old Schwartz equation. Our creatinine values were obtained with Jaffe methods, so we expected the old Schwartz equation (based on Jaffe methods) to outperform the new Schwartz equation (based on enzymatic methods), but this did not seem to be the case and likely relates to the fact that the old Schwartz equation overestimates eGFR in children [20]. Third, though the new Schwartz equation and the India equation appeared similar, the new Schwartz equation is more commonly used and would allow for greater comparisons worldwide.

### Limitations

We powered our study to evaluate the incidence of AKI in this pediatric trauma population, and evaluation of risk factors and AKI definitions were secondary aims. Thus, risk factors that were significant certainly deserve further exploration, but those that were not significant should not be excluded from future studies or clinical consideration as we had limited power to detect differences given our small sample size (ie, malaria, malnutrition, drinking water, etc). In addition, due to lack of baseline creatinine values we cannot be certain that the creatinine abnormalities seen in this study are directly caused by trauma. However, AKI was primarily associated with trauma-related factors (burn injuries, multiple injuries), not other potential confounders (malaria, malnutrition, sickle cell). The interesting factor that certainly could be a confounder was the finding of herbal remedies associated with higher risk of AKI, and this needs to be further investigated.

While we did not reach our goal enrollment due to logistical and financial constraints, we enrolled > 100 patients, making this the second largest pediatric trauma study worldwide to evaluate AKI. Given the lack of electronic medical records and paucity of data in paper records, we collected all data prospectively to maximize accurate data collection. As such, we also could not further hypothesize to the etiology of the AKI (pre-renal, ATN, multifactorial, etc), only whether or not renal dysfunction was present.

A large number of eligible patients were missed due to logistical reasons (*n* = 270). We compared patients missed with those enrolled and found no significant differences in gender, age, or mechanism of trauma. Based on our enrollment criteria, we did miss enrolling more patients who presented on weekends. We expected mechanism of trauma to differ, but it did not; it is possible that patients with more severe injuries presented on the weekends. Therefore, we may have underestimated the incidence of AKI in this population. In addition, patients who died prior to or shortly after arrival were not included in this study and may have also led to underestimations in our estimate of the AKI incidence.

This was the first pediatric AKI study in African trauma patients, yet due to resource limitations we were only able to obtain at most two creatinine values on a patient and no urine output, which may have led to some missed cases of AKI. To overcome the lack of consensus on estimating a baseline creatinine, we evaluated several methods to investigate a potential optimal method for this setting. Ideally, our findings should be confirmed with datasets that contain known baseline creatinine values, but to our knowledge these are not widely available in sub-Saharan Africa. Estimating a child’s baseline creatinine is an area that deserves more research to further standardize the AKI definition and subsequent diagnostic management plans in limited resource settings.

## Conclusion

AKI occurs in about 10% of admitted pediatric trauma patients in Malawi, though the incidence varies depending on how one estimates the baseline creatinine. In this patient population, the new (bedside) Schwartz equation appeared to perform best. A high index of suspicion for AKI should be considered for pediatric trauma patients, as early management may improve outcomes. Strikingly, those with AKI were 7 times more likely to die as compared to those without AKI. Further research should confirm these findings and explore if early identification of AKI in pediatric trauma patients can be combined with management strategies to decrease mortality in this high-risk population.

## Data Availability

The datasets generated and/or analysed during the current study are not publicly available due to protection of privacy but are available from the corresponding author on reasonable request.

## Abbreviations

AKI: Acute Kidney Injury
CI: Confidence Interval
eGFR: estimated Glomerular Filtration Rate
IQR: Interquartile Range
KCH: Kamuzu Central Hospital
KDIGO: Kidney Disease Improving Global Outcomes
RR: Relative Risk
UNC: University of North Carolina
